# Lignocellulose depolymerization occurs via an environmentally adapted metabolic cascades in the wood-rotting basidiomycete *Phanerochaete chrysosporium*

**DOI:** 10.1002/mbo3.228

**Published:** 2014-12-03

**Authors:** Jin Seop Bak

**Affiliations:** Department of Chemical and Biomolecular Engineering, Advanced Biomass R&D CenterKAIST, 291 Daehak-ro, Yuseong-gu, Daejeon, 305-701, Republic of Korea

**Keywords:** Bioconversion, biofuels, lignocellulose, metabolic networks, *Phanerochaete chrysosporium*, systems biology

## Abstract

Plant biomass can be utilized by a lignocellulose-degrading fungus, *Phanerochaete chrysosporium*, but the metabolic and regulatory mechanisms involved are not well understood. A polyomics-based analysis (metabolomics, proteomics, and transcriptomics) of *P. chrysosporium* has been carried out using statistically optimized conditions for lignocellulolytic reaction. Thirty-nine metabolites and 123 genes (14 encoded proteins) that consistently exhibited altered regulation patterns were identified. These factors were then integrated into a comprehensive map that fully depicts all signaling cascades involved in *P. chrysosporium*. Despite the diversity of these cascades, they showed complementary interconnection among themselves, ensuring the efficiency of passive biosystem and thereby yielding energy expenditure for the cells. Particularly, many factors related to intracellular regulatory networks showed compensating activity in homeostatic lignocellulolysis. In the main platform of proactive biosystem, although several deconstruction-related targets (e.g., glycoside hydrolase, ureidoglycolate hydrolase, transporters, and peroxidases) were systematically utilized, well-known supporters (e.g., cellobiose dehydrogenase and ferroxidase) were rarely generated.

## Introduction

With the world increasingly facing environmental instability and an emerging energy crisis, forest bioenergy production (especially bioethanol) from renewable feedstock has been actively reviewed as an alternative solution to the dependency on fossil fuels (Hudiburg et al. 2011). To effectively supply cellulosic bioethanol, the conversion of recalcitrant biomass into a fermentable carbon chain is a key process (i.e., substrate pretreatment) (Chen and Dixon 2007; Sanderson 2011).

Recent studies of biomass pretreatment have focused on novel methodologies using a microbiological-based bioconversion system to replace the conventional physicochemical processes with more environmentally friendly ones (Menon and Rao 2012). In particular, white-rot basidiomycete *Phanerochaete chrysosporium* is one such species of fungus that is most extensively studied for its ability to degrade biomass feedstock. The Joint Genome Institute of the US Department of Energy recently released the assembly and model of the *P. chrysosporium* genome (http://jgi.doe.gov/genome-projects/); however, the detailed mechanisms through which this fungus regulates lignin degradation are still not fully understood. Despite this, no research in the past has attempted to understand the detailed interactions in microbial signaling networks at omics level. Furthermore, no previous studies have applied to statistically optimize before process evaluation to verify major factors in simultaneous whole-cell metabolisms.

In this research, a combined bottom-up evaluation, based on polyomics and target optimization, has been attempted to elucidate the metabolic and regulatory systems involved in lignocellulolysis by *P. chrysosporium*. In particular, target optimization was used to elucidate the important interconnected factors in the hydrolysis cascades, and rearranged vast regulator profiles into specific information about lignocellulolytic factors. An optimized understanding of the entire cascades is provided to elucidate the signaling interconnections of this fungus, and thus to better identify its substrates. Furthermore, this combined approach to rapidly describe cellular networks defines a useful discipline within the field of downstream bioprocesses.

## Materials and Methods

### Substrates and cultivation conditions

Lignocellulosic rice straw (RS) as a model compound of plant biomass was harvested from Korea University Farm (Deokso, Korea). After the preprocessing steps (e.g., washing, milling, and drying; Supporting Information), processed RS was used as the starter material for deciphering lignocellulolytic pathways in fungal biosystem. *Phanerochaete chrysosporium* ATCC 32629 was used. The fungal conidia were harvested from the periphery of 7- to 15-day-old cultures that were grown on potato/dextrose/agar plates using a sterile spatula. The spores were inoculated at 2.7 × 10^6^ spores/mL, and then incubated at 30°C with shaking at 150 rpm for 72 h. The spore concentration was checked by suspending conidia in 0.85% (w/v) sterile saline and then counting the spores in a cell counting chamber (Neubauer; Marienfeld, Germany). After the addition of 10.0 g RS, *P. chrysosporium* was cultured in 400 mL of optimized fungal medium (Bak et al. 2009a) containing 1% (w/v) of glucose as initial carbon source at 29°C and 150 rpm for 30 days. Further details regarding the medium optimization are provided in Supporting Information. No substrate was added to the control cultures.

### Practical evaluation of optimized biosystem

Analytical samples were collected from the culture broths (with reproducibility based on process evaluation), and then passed through a 0.2-*μ*m PVDF filter (Whatman, Brentford, UK). The total protein concentration in each broth was then reproducibly checked using a BCA protein assay kit (Pierce, Rockford, IL). Based on broadly accepted methods (Supporting Information), the cellular activities of key targets involved in lignocellulose biodegradation were simultaneously checked during the long-term fermentation. The generation of peroxidative radicals was confirmed using the OxiSelect fluorometric tool (STA-344; Cell Biolabs, San Diego, CA) (http://www.cellbiolabs.com/). These mixtures were incubated for 30 min in the dark, and then fluorescence was checked with excitation at 530 nm and emission at 590 nm. According to the standard analytical protocols (http://www.nrel.gov/biomass/analytical_procedures.html), the change of key components (cellulose, hemicellulose, and lignin) of RS substrate was analyzed based on a dry weight basis (per 100 g biomass). Furthermore, the % theoretical yields (here monomeric sugar and ethanol) as index of scale-up (or downstream process) evaluation were analyzed based on the standard protocols. Further details are provided in Supporting Information. Additionally, based on a modified version of the θ–2θ method (Bak et al. 2009b), the exposure index of crystalline substrates was predicted using a powder diffractometer (Bruker D5005, Karlsruhe, Germany).

### Simultaneous transcriptome analysis

After six biological replicates of the cultures, *P. chrysosporium* mycelia from 15-day cultures were collected by filtering through a 0.2-*μ*m filter (Whatman) and snap-freezing in liquid nitrogen. Total RNA was isolated from the fungal pellets using 0.2 g zirconia/silica beads (0.5–1 mm; Biospec Products, Bartlesville, OK) and a Mini Beadbeater (Biospec) with 1 mL TRIzol reagent (Invitrogen, Carlsbad, CA), according to the manufacturer's recommendations. Finally, the frozen samples are stored in a deep-freeze cabinet (−80°C). Further details are provided in Supporting Information. Sequentially, cDNA was synthesized using the Superscript™ II RT-PCR System (Invitrogen, Karlsruhe, Germany) according to the manufacturer's instructions. Hybridization was performed with 5 *μ*g labeled sample per Custom Array 12K microarray (CombiMatrix Corporation, Mukilteo, WA). As shown in Table S1, based on the tendency of the expressed regulation (upregulated or downregulated), the reliability of the array was confirmed with the quantitative real-time PCR results of predominant five targets. Further details are provided in Supporting Information. After the statistical data preprocessing (Supporting Information), hierarchical clustering analysis was performed to organize significant genes into functional clusters (Eisen et al. 1998). PermutMatrix ver. 1.9.3 (http://www.atgc-montpellier.fr/permutmatrix/) was the graphical analysis software used to determine changes in the patterns of expression across groups (Caraux and Pinloche 2005).

### Proteome mapping analysis

Similar to the transcriptome analysis, fungal mycelia (from 15-day cultures) were collected by filtering and snap-freezing. The treated samples are stored in a deep-freezer. After protein extraction from *P. chrysosporium* pellets based on the standard procedures (Supporting Information), reference mapping of proteome was performed using two-dimensional gel electrophoresis to evaluate the quantitative patterns of changing proteins under RS, as compared to the control. The changed spots were identified by sequencing, and peptide mass fingerprinting based on the public database. Further details are provided in Supporting Information. After six biological replicates, statistical improvement of all encoded proteins was carried out using SAS program ver. 9.2 (SAS Institute, Cary, NC) and SigmaStat 3.5 (Systat Software, San Jose, CA). The hierarchical analytical method was used for complete linkage of the functional proteins.

### Analysis of metabolome distribution

A quadrupole-type GC-MS (5975 model; Agilent Technologies, Waldbronn, Germany), which contains a 7890 GC system (Agilent Technologies) and a DB-5MS (J&W Scientific, Folsom, CA), was used for the identification and quantification of cellular metabolites. Further details regarding the operation and sample pretreatment are provided in Supporting Information. After eight biological replicates, the significance of abundance changes for the metabolites in each culture was confirmed using the paired *t*-test. The differences among groups were determined using the unpaired *t*-test and analysis of variance (ANOVA). The statistical tests for metabolome data were performed using both SAS and SigmaStat. Functional metabolic groups were divided by the hierarchical methodology.

### Determining cellular pathways from multiple omics

First, interconnected pairs of genes (or proteins) and metabolites are identified by mass balance (based on fold-change and extracellular activity) using a known genome-wide metabolic pathway. Next, the data points on metabolite, protein, and transcript changes are displayed in a vast map of biochemical networks. Detection of a series of upregulated targets and related factors positively hints toward a mainstream pathway. On the other hand, downregulation of the factors suggests either the presence of a bottleneck or that the purported pathway is less favorable than alternative routes. Further details are provided in Supporting Information.

## Results and Discussion

### Functional categorization of predominant biodegradation targets

**Figure 1:**
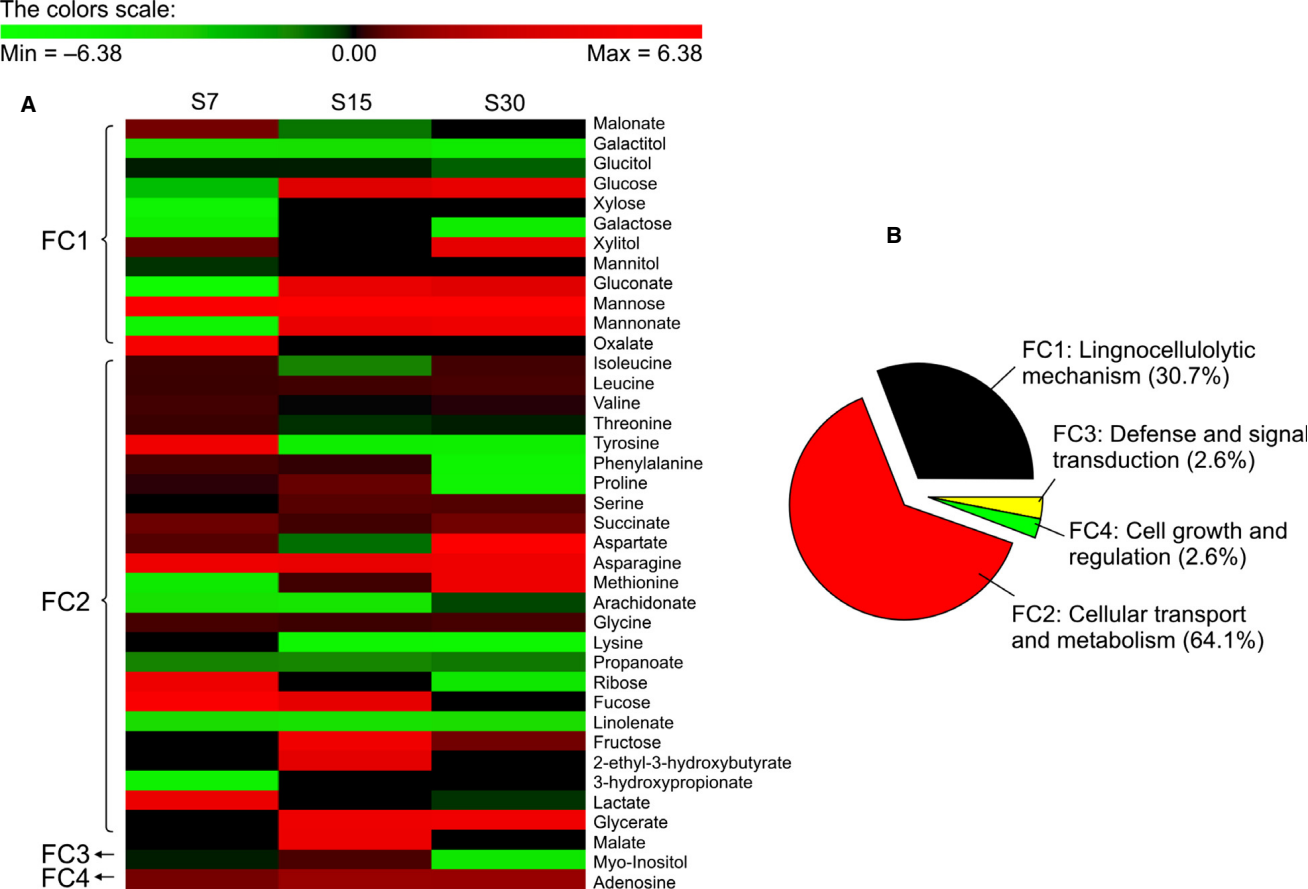
Metabolomic profiling of *Phanerochaete chrysosporium* grown with rice straw versus control. (A) Expression profiles from culture grown on RS for 7 (S7), 15 (S15), and 30 (S30) days. Hierarchical clustering of 39 metabolites showing considerable variation in expression with |fold| > 2 and 0.01 ≤ *P *<* *0.05 in RS culture. The color scale reflects the logarithmic unit in comparison with the control. (B) Functional clustering of the 39 metabolites was grouped with biochemical functions released by the National Institute of Standards and Technology database.

**Figure 2:**
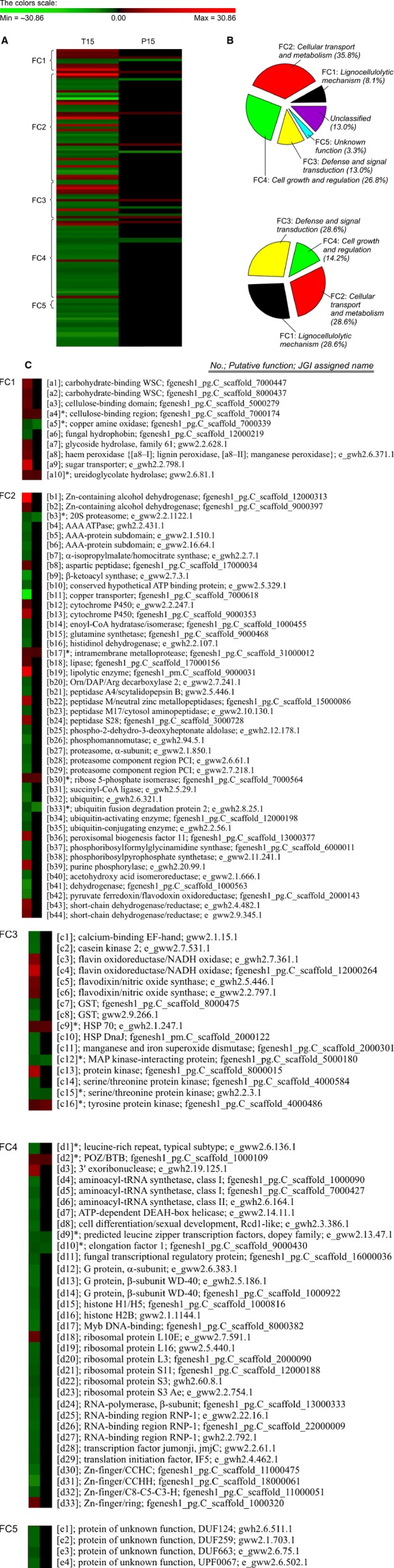
Combined upstream profiling of optimized *Phanerochaete chrysosporium* grown with rice straw. (A) Hierarchical clustering of noteworthy targets (123 genes and 14 encoded proteins) showing considerable differences in expression with |fold| > 2 and *P *<* *0.05 in RS culture. The encoding proteins are marked with asterisks (*). Lanes T15 and P15 are transcriptome and proteome expression profiles, respectively, from culture grown on RS for 15 days. (B) Functional cluster classification of the 123 genes (upper) and 14 proteins (bottom) based on the putative molecular functions declared by the US Department's Joint Genome Institute. (C) Putative functions of the factors based on the Joint Genome Institute's public database.

**Figure 3:**
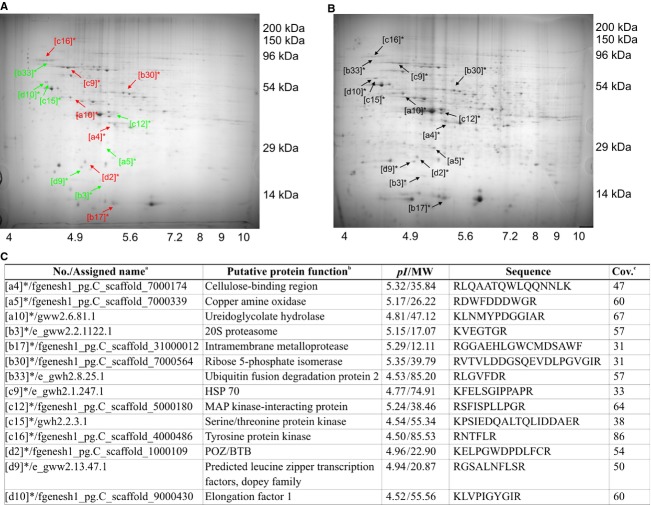
Protein expression profiles of *Phanerochaete chrysosporium* biosystem. The fungus was cultured for 15 days on (A) rice straw. The (B) control was grown without these substrates. Fourteen spots on the two-dimensional gel electrophoresis represent intracellular target proteins with |fold| > 2 and *P *<* *0.05, that is, showing significantly higher (*red*) or lower (*green*) expression levels compared to the corresponding proteins in control. (C) Identification of the intracellular proteins by MALDI/MS/MS peptide mass fingerprinting. ^a,b^The names and functions of selected genes encoding proteins of *P. chrysosporium* were assigned based on US Department's Joint Genome Institute. ^c^Sequence coverage (%) in peptide mass fingerprinting.

After biodegradation of *P. chrysosporium* under optimized conditions for 30 days with RS or no substrate (control), the overall metabolomic profiles of *P. chrysosporium* were examined (Fig.1). Based on the National Institute of Standards and Technology downstream database (http://www.nist.gov/), 39 target metabolites (|fold| > 2 and 0.01 ≤ *P *<* *0.05), which were subsequently categorized into four functional clusters (FC1–4) were identified (Fig.1B). Especially, the predominant metabolites are summarized in Table1. Next, upstream profiling after 15 days (% actual maximum of glucose) of biodegradation to verify the downstream data was carried out (Fig.2). Significant 123 genes (|fold| > 2 and *P *<* *0.05) out of the functionally annotated 5621 genes that were consistently upregulated or downregulated with RS substrate treatment were discovered. Among over 200 spots in each proteome map that were observed, significant 14 encoded proteins (|fold| > 2 and *P *<* *0.05) showed distinct expression patterns in the RS samples compared to the control (Fig.3). Similar to the metabolomics study earlier, upstream targets were also classified by hierarchical clustering (FC1–4 and ungrouped; Fig.2) and were identified using upstream database from the US Department of Energy Joint Genome Institute (http://genome.jgi.doe.gov/) (Table S1). The predominant genes are summarized in Table2.

**Table 1:** List of significant metabolites correlated with lignocellulolytic metabolic cascade during optimized *Phanerochaete chrysosporium* fermentation with rice straw

			|Fold change|	
Target metabolite	Day^1^	Change in expression^2^	RS: control^3^	*P*-value^4^
Galactitol	15–30	Downregulated	3.7–4.2	0.01–0.00
Glucose	15–30	Upregulated	3.6–4.1	0.02–0.00
Xylose	7	Downregulated	4.8	0.00
Galactose	30	Downregulated	4.4	0.00
Xylitol	30	Upregulated	4.1	0.00
Gluconic acid	15–30	Upregulated	4.2–3.7	0.00–0.01
Mannose	15–30	Upregulated	6.3–6.4	0.00–0.00
Mannonic acid	15–30	Upregulated	4.2–4.4	0.00–0.00
Oxalic acid	15	Downregulated	3.8	0.00
Arachidonic acid	15	Downregulated	4.0	0.00
Linolenic acid	15–30	Downregulated	3.7–3.5	0.00–0.01
Fructose	15	Upregulated	4.6	0.00
2-ethyl-3-hydroxybutyrate	15	Upregulated	3.9	0.00
3-hydroxypropionic acid	7	Downregulated	4.7	0.00
Glyceric acid	15–30	Upregulated	4.5–4.6	0.00–0.00
Malic acid	15	Upregulated	4.3	0.00

1 Culture period prior to analysis when the significant change was observed.

2 Relative expression of RS compared to that of control (no substrate).

3 Relative fold change ratio of the targets between RS and control.

4 *P*-value was calculated using paired *t*-test.

**Table 2:** List of significant genes correlated with lignocellulolytic regulatory and metabolic system during optimized *Phanerochaete chrysosporium* fermentation with rice straw

			|Fold change|	
Target gene	Putative function^1^	Change in expression^2^	RS: Control^3^	*P*-value^4^
[a1]	Carbohydrate-binding WSC	Upregulated	4.5	0.01
[a2]	Carbohydrate-binding WSC	Upregulated	2.6	0.05
[a3]	Cellulose-binding domain	Upregulated	2.9	0.05
[a4]	Cellulose-binding region	Upregulated	2.0	0.05
[a5]	Copper amine oxidase	Downregulated	2.1	0.03
[a7]	Glycoside hydrolase	Upregulated	2.8	0.00
[a8]	Haem peroxidase	Upregulated	3.8	0.01
[a9]	Sugar transporter	Upregulated	14.7	0.00
[a10]	Ureidoglycolate hydrolase	Upregulated	2.2	0.05
[b1]	Zn-alcohol dehydrogenase	Upregulated	30.7	0.00
[b2]	Zn-alcohol dehydrogenase	Upregulated	3.3	0.02
[b4]	AAA ATPase	Downregulated	3.3	0.00
[b11]	Copper transporter	Downregulated	21.5	0.00
[b12]	Cytochrome P450	Upregulated	2.5	0.02
[b13]	Cytochrome P450	Upregulated	8.8	0.00
[b43]	Short-chain dehydrogenase/reductase	Upregulated	5.8	0.00
[b44]	Short-chain dehydrogenase/reductase	Upregulated	4.3	0.00
[c1]	Calcium-binding EF-hand	Downregulated	3.2	0.00
[c3]	Flavin oxidoreductase/NADH oxidase	Upregulated	5.2	0.00
[c4]	Flavin oxidoreductase/NADH oxidase	Upregulated	11.8	0.00
[c5]	Flavodoxin/nitric oxide synthase	Upregulated	3.9	0.00
[c6]	Flavodoxin/nitric oxide synthase	Upregulated	3.2	0.04
[c7]	Glutathione *S*-transferase	Downregulated	2.3	0.01
[c8]	Glutathione *S*-transferase	Downregulated	3.5	0.00
[c11]	Manganese and iron superoxide dismutase	Downregulated	3.2	0.00
[c12]	MAP kinase-interacting protein	Downregulated	2.2	0.05
[c13]	Protein kinase	Upregulated	8.8	0.00
[c14]	Serine/threonine protein kinase	Downregulated	2.5	0.05
[c15]	Serine/threonine protein kinase	Downregulated	2.3	0.00
[c16]	Tyrosine protein kinase	Upregulated	2.1	0.05
[d2]	POZ/BTB	Upregulated	2.5	0.05
[d31]	Zn-finger/CCHH	Downregulated	5.4	0.00

1 The putative functions of the selected genes of *P. chrysosporium* were assigned based on the US Department's Joint Genome Institute database.

2 Relative expression of RS compared to that of control (no substrate).

3 Relative fold change ratio of the targets between RS and control.

4 *P*-value was calculated using paired *t*-test.

### Nonspecific simultaneous metabolic regulation based on target optimization

Broadly, the simplification of fundamental metabolic cascades in microbial cells is expected as a means to actively maintain the self-defense mechanism from severe environments. Similarly, the lignocellulolytic bioconversion induced by *P. chrysosporium* is thought to require the overexpression and optimization of major factors (especially lignin-metabolizing enzymes and their precursors). In particular, systematic cascades through the selective activation of powerful targets (especially manganese peroxidase [MnP]) were imperative in order to fulfill the heavy energy expenditure of biodegradation-related processes, in turn optimizing the overall balance of *P. chrysosporium* biosystem (Figs.1 and 2). Despite the selective maximization of targets, the biosystem do not still rule out minor factors (∼80% of total) which are central-mediated functions of certain signals (e.g., growth and stress response), on individual metabolic fluxes. This implies that perturbations in the regeneration rate of regulators are disseminated through the global metabolic network.

**Figure 4:**
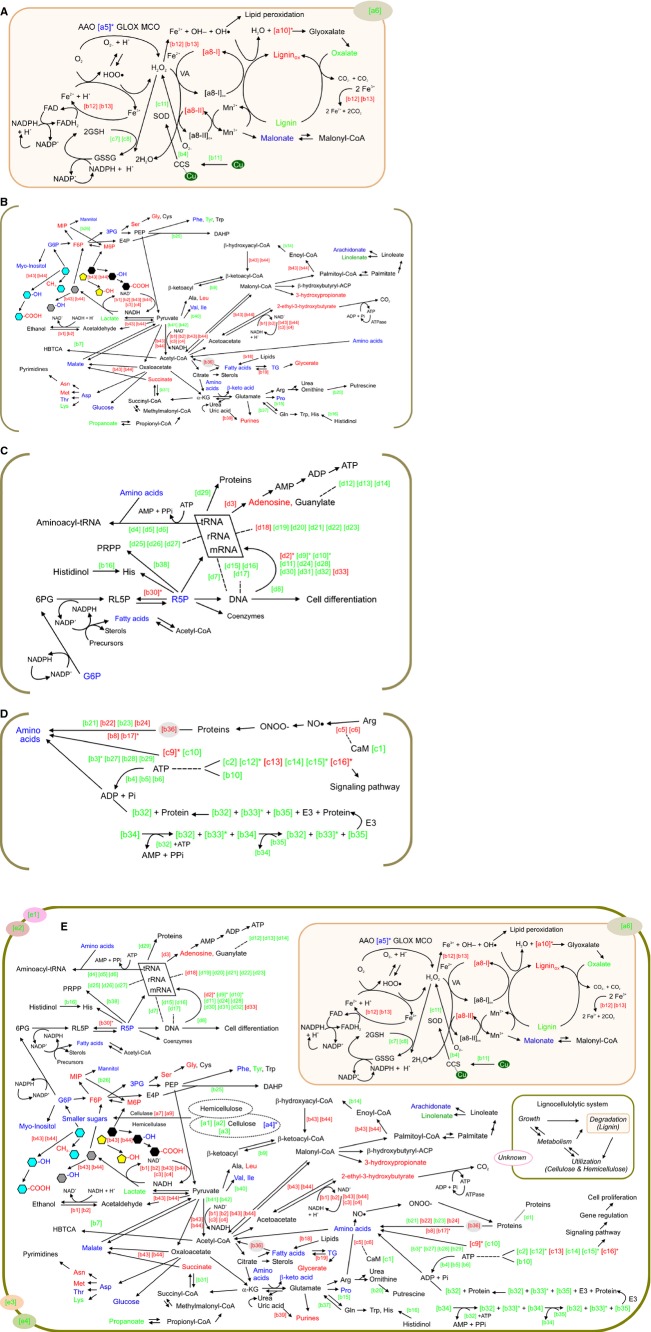
Proposed cellular system of lignocellulolytic biodegradation based on the integration of multi-omics profiles of *Phanerochaete chrysosporium* grown under optimized degradation condition with rice straw (consisting of both amorphous and crystalline polymers) as a substrate. (A) Ligninolytic cascade by reactive oxygen species-induced mechanisms. (B) Cellular metabolism for energy regulatory and maintenance. (C) Growth and regulatory system. (D) Cell-to-cell signaling and defense and ubiquitin proteasome system. (E) Overall lignocellulolytic biosystem by *P. chrysosporium*. Predominant regulatory genes (|fold| > 2 and *P *<* *0.05) at 15 days are indicated in *red* (upregulated), *green* (downregulated), and *blue* (either upregulated or downregulated) based solely on the transcriptome level. The ones confirmed by both transcriptomic and proteomic data are marked with asterisks (*). Metabolites that show considerable expression levels (|fold| > 2 and 0.01 ≤ *P *<* *0.05) compared to the control at later time points (15–30 days) were colored *red* (up), *green* (down), and *blue* (either up or down). The dotted line denotes specific binding interaction. Putative functions of all targets are shown in Figure2. The *black*, *gray*, and *sky*-*blue* hexagons represent mannose, galactose, and glucose, respectively, and yellow pentagon represents xylose. 3PG, 3-phosphoglycerate; 6PG, 6-phosphogluconate; α-KG, α-ketoglutarate; CaM, calmodulin; CCS, copper chaperone for SOD; CoA, coenzyme A; DAHP, 3-deoxy-d-arabino-hept-2-ulosonate 7-phosphate; E3, ubiquitin-protein ligase; E4P, erythrose 4-phosphate; F6P, fructose 6-phosphate; G6P, glucose 6-phosphate; HBTCA, 2-hydroxybutane-1,2,4-tricarboxylate; M1P, mannose 1-phosphate; M6P, mannose 6-phosphate; NAD, nicotinamide adenine dinucleotide; NADH, reduced form of NAD; NADP, nicotinamide adenine dinucleotide phosphate; NADPH, reduced form of NADP; ox, oxidized form; PEP, phosphoenolpyruvate; PRPP, phosphoribosylpyrophosphate; R5P, ribose 5-phosphate; RL5P, ribulose 5-phosphate; SOD, superoxide dismutase; TG, triacylglycerols; VA, veratryl alcohol.

Briefly, the interconnected lignocellulolytic networks of self-regulated metabolisms in *P. chrysosporium* could be divided into the following four steps (Fig.4): (1) growth and functional metabolism by which cells grow using the initially added nutrient sources along with lignocellulosic substrates; (2) direct or indirect reactive oxygen species (ROS)-mediated deconstruction of the lignin polymer; (3) production of metabolic intermediates from exposed polysaccharides; (4) synergistic networking system by regulatory factors related to intracellular and extracellular signaling; and rotation of the above procedures without the supply of additional substrates. Interestingly, the expression of lignocellulose-degrading enzymes during *P. chrysosporium* fermentation continually produced and upgraded their metabolic products regardless of the absence of lignin-related substrates (i.e., negative control; Figs.1 and 2). These results imply that initially added fermentable sugars (or rapidly limited nitrogen sources) function as inducers of ligninolytic and cellulolytic genes in *P. chrysosporium*, which has been confirmed in other cellulolytic fungi (Bak 2014; Znameroski et al. 2012). The extracellular activation of predominant targets indicated that cellulosic materials must be involved during the initial stages of the lignocellulolytic bioprocess.

### Peroxidative attack for open access: ligninolytic cascades

Open *P. chrysosporium* system suggests that ROS-mediated program ([a5]*, [a10]*, [c7], [c8], [c11], ferric-oxalates, and Mn(III)-malonate) and mono-oxygenation and hydroxylation ([b12] and [b13]) are essential for providing cells with the capability of lignin degradation (Figs.1A, 2A, and 4A and B2 and 3). Unlike previously well-known concepts (from the white-rot fungal mechanism) relevant to the peroxidative cascades (via free radicals) of lignin complexes (Cullen and Kersten 2004), the expressions of useful ligninolytic enzymes (e.g., ferroxidase, aryl-alcohol oxidase [AAO], glyoxal oxidase [GLOX], and catalase; |fold| < 2) did not vary predominantly (probably due to the process optimization based on the lignocellulolysis efficiency); however, their activations could be predicted from the induction of biomarkers (e.g., benzaldehydes; Fig.1A) and the actual cellular activity (Table3). Remarkably, because the expanded platform (i.e., open form of cellulosic structures) of the lignocellulolytic fermentation may happen mostly outside the cell, the differences of extracellular depolymerization (e.g., % theoretical yields; Table4) can be coincided with the real state of *P. chrysosporium* biosystem. In the real large-scale system, the action of the extracellular enzymes was also confirmed by the loss (approximately 22.0%) of lignin content. Interestingly, upon comparing with a previous report (nonoptimization and small-scale fermentation; Bak et al. 2009a), powerful activities in a similar range of core enzymes (especially MnP, lignin peroxidase, AAO, and GLOX) during a 15-day fermentation. This is probably due to the real-time metabolic conversion of homeostatic energy regulation in the *P. chrysosporium* cascades, similar to that suggested in the other ligninolytic biosystem (Bak et al. 2010).

**Table 3:** Bottom-up analysis of core lignocellulolytic enzymes in optimized *Phanerochaete chrysosporium* biodegradation after 15 days

Type	ROS^1^ (mmol/L)	Extracellular activity of key enzymes (U/L or U/mg protein)			
		Lignin peroxidase Manganese peroxidase	Glyoxal oxidase Aryl-alcohol oxidase Ferroxidase^2^	*β*-glucosidase Cellobiose dehydrogenase Xylanase	Manganese superoxide dismutase Catalase P450-oxidoreductase Glutathione^3^
Biodegraded	<0.02	700–900	∼500	∼130	<80 U/mg
		1200–1800	∼200	∼60	∼200 U/mg
			<50	∼480,000	<40 U/mg
					<2.8
Untreated	–	–	–	–	–
		–	–	–	–
			–	–	–
				–	–
					–

1 Reactive oxygen species; here H_2_O_2_.

2 It contains the activity of multicopper oxidase.

3 Glutathione/Glutathione disulfide ratio.

**Table 4:** Industrial mass balance in optimized *Phanerochaete chrysosporium* biosystem after 15 days

Type	Total solid (dry wt. basis)	Change of core components (g/L) (before/after)			Fermentable sugar (g/L)	Index of evaluation	
		Lignin	Cellulose (g glucan)	Hemicellulose (g xylan)	Glucose^1^	% Sugar^2^	% Ethanol^3^
Biodegraded	25.0 g RS	5.0/3.9	8.9/7.5	2.7/2.1	<0.25	∼67	∼65
Untreated	25.0 g RS	5.0/5.0	8.9/8.9	2.7/2.7	–	∼27	∼30

1 Soluble glucose from the cellulosic substrates during the SSF.

2 % Theoretical maximum glucose yield from the enzymatic hydrolysis.

3 % Theoretical maximum ethanol yield from the SSF.

In detail, Mn(III)-malonate, which is oxidized by activated peroxidases (especially MnP) as a biodegrading initiator, is reduced to Mn(II) by oxidized recalcitrant polymeric chains (Fig.4A). In particular, the coregulation of peroxidase/ferric-oxalate (or FeCl_3_) signals may play a vital role for the spontaneous metabolic trigger of the ligninolytic process, similar to the other white-rot fungal biosystem (Mäkelä et al. 2014). Especially, the posttranscriptional control by manganese is directly cooperated in the activation of MnP under the nitrogen starvation (Kamei et al. 2008). Although the MnP devices (especially versatile or short-module) of extracellular peroxidation have recently focused on biodegradation events by white-rot fungi (Salame et al. 2014); however, this platform was not induced in our system. Furthermore, lignin peroxidases have depolymerization capability toward exposed recalcitrant substrates (e.g., arylamines and aromatic thiols), and they may be indirectly involved in conserving carbon metabolism (sugar recovery based on crystallinity; Tables3 and 4). Simultaneously, to complement the incomplete deconstruction cascades (and further metabolic homeostasis), various redox-potential balancers (containing a flavin adenine dinucleotide or heme residue) based on the antioxidant mechanism can participate in ligninolytic network (Fig.4E and F3). In particular, intracellular metabolic connection via mono-oxygenation (especially GLOXs, AAOs, ferroxidases, superoxide dismutases [SODs; [c11]], and P450-oxidoreductases) is as important as the extracellular ligninolytic regulators. Furthermore, the factors [b11] and [b4], related to intracellular trafficking, were predicted to be essential to the delivery system, and copper chaperones for SOD were found to be part of a membrane shuttle system (O'Halloran and Culotta 2000). Furthermore, the above-mentioned coregulators could dependently participate in the metabolic feedback of electron acceptors (e.g., ferric ions, oxalates, malonates, and molecular oxygen) and the downstream cascade of biodegradable polymers to CO_2_ and H_2_O (Figs.1A and 4A). Resultantly, fungal-induced ring-cleavage mechanism (by biocatalysts) could directly convert lignocellulosic substrate into both depolymeric fragments and their derivatives (e.g., dicarboxylic acids) during the cell-wall disruption. Additionally, the naturally occurring biominerals (especially carbonate and phosphate) produced in turn participate in further ligninolytic processes. For reference, the presence of aspartate (downregulation of day 15; Fig.1A) may induce the positive control of metabolic biomineralization by an improvement of enzyme kinetics (Piana et al. 2007). Additionally, the activation of hydrophobic hydrophobins ([a6]) may contribute to aerobic fermentation by assisting in the collaboration of extracellular hydrolases (Wessels 1994). Lastly, the limitation (downregulation of day 15; Fig.2A) of cell-bound unknown proteins ([e1], [e2], [e3], and [e4]) may also contribute to stable biodegradation, which is a driving force for the production of ligninolytic controllers.

### Cellulolytic biodegradation: regulation and extracellular metabolism of CAZys

In activated wood-degrading biosystem, a cocktail of carbohydrate-active enzymes (CAZys; especially carbohydrate-binding modules [CBMs; [a1], [a2], [a3], and [a4]*], glycoside-hydrolases [GHs; [a7]], cellobiose dehydrogenase [CDH], *β*-glucosidase, and xylanase) showed the constitutive enhanced expression patterns throughout the entire *P. chrysosporium* cascades (Figs.2A and 4E and F and Table 2 and 3). In particular, the collaboration between extracellular CAZys are known to couple with CBMs via glycosylated connections (by GHs) to construct active complex modules, especially in the presence of biocatalysts (especially metal chelators; Harris et al. 2010). In the other biosystem (Vanden Wymelenberg et al. 2010; Fernandez-Fueyo et al. 2012), GHs in particular have also been shown to selectively enhance the catalytic effects of cellulolytic (or hemicellulolytic) enzymes. Similar to previously reported concept (Resch et al. 2013), in the CBM-mediated deconstruction system by *P. chrysosporium*, the extracellular activity of fungal cellulosomes with high GH dependency are advantageous for the induction of monomeric sugars (e.g., remained glucose, <0.25 g/L; Table4) rather than for oligomers. In a view of cellulolytic enhancing regulation, the oxidoreductive coupling of copper-dependent GHs (especially family 61) is important, and the interactions between GHs and cellulases (especially CDH) have been reported to dramatically increase the extracellular breakdown of lignocellulosic materials (Langston et al. 2011; Quinlan et al. 2011).

Interestingly, when compared with the activities of ligninolytic targets, the predominant CAZys may exhibit very effectiveness (Fig.2 and Table3). Although several CAZy factors (especially CDH, *β*-glucosidase, and xylanase) did not differ from the control in their upstream patterns, their activity was altered in the extracellular system. For example, the extracellular hemicellulolytic activity of xylanase reached a maximum at 480,000 IU/L after 15 days of actual biosystem. Unexpectedly, unlike the previous upstream studies (Sato et al. 2009; Fernandez-Fueyo et al. 2012), well-known cascade (especially by CDH and *β*-glucosidase) in final step of cellulolysis had a very low influence. Furthermore, in downstream properties, when cellulose/hemicellulose cultures (Bao et al. 1994; Vanden Wymelenberg et al. 2010) processed, the extracellular activities of both *β*-glucosidase and CDH was activated as high values compared to those of current *P. chrysosporium* system (i.e., lignocellulose culture; Table3). These results were also confirmed by the loss of the crystalline polymers (especially xylan and glucan) of RS as well as the enhancement of both enzymatic hydrolysis (64.0% of maximum) and fermentability (65.0% of maximum) during simultaneous saccharification and fermentation (Table4). For reference, this program was superior to conventional microbiology-based programs, in terms of cost saving (<50% of glucose maximum and <40% of ethanol maximum) (Dashtban et al. 2009; Shi et al. 2009). However, the yields were not higher than those of plant biomass (>71% of glucose maximum) pretreated using conventional physicochemical programs (Kim et al. 2002; Ko et al. 2009). Consequently, the open *P. chrysosporium* cascade is a cell-wall-loosening process, based on the bioaccessibility between proactive CAZys and heterogeneous polymeric chains (i.e., crystalline or amorphous).

In the downstream signaling, extracellular metabolic factors, included hydrolysates (e.g., monomeric sugars) and their derivatives (e.g., carboxylic acids and sugar alcohols), showed progressively stronger regulation in the presence of RS than that in the control culture (Figs.1A and 4E and F1). In particular, the byproducts (especially glucose, mannose, and xylitol) from CAZys predominantly activated (>4.1-fold) after 15 days. The exceptions among these were a few intermediates, including malonate and oxalate, which were previously mentioned. After the biodegradation under the severe condition, released sugars must be involved in cellular metabolic system (without exception) in order to maintain an instinct for survival. Therefore, proactive sensors have evolved as metabolic transporters (especially [a9]; Fig.2A) to perceive their targets, harboring signaling domains at the plasma membrane. For example, sugar metabolisms are highly regulated by sugars and specific proteins (such as sucrose transporter [SUT] and monosaccharide transporter [MST]; André 1995; Ayre 2011) at the genome level.

### Energy regulatory and cellular maintenance

Many factors related to the Kreb's cycle were found to be complementarily regulated during *P. chrysosporium* lignocellulolysis (regardless of culture periods), probably due to their role in anaplerosis and cataplerosis (Figs.1A and 4B and C and Table1). However, most key factors involved in glycolysis were generally upregulated by pretreatment after 15 days. In particular, the activation of mannitol cascade (downregulation after 7 days) plays a part in the regeneration of NADP(H). Intrametabolic signaling mechanisms directly underpin the reducing powers of glycolytic turning points via certain dehydrogenases (especially [b1] and [b2]) and their cofactors (NAD[H] and ferredoxin). Similar to the previous results (Rieble et al. 1994; Shary et al. 2008), *β*-ketoadipate system, which is responsible for maintaining minerals, was spontaneously interconnected based on NAD(P)H and cytochrome P450 ([b12] and [b13]). Likewise, Schiff base products were decreased via dehydrogenases/reductases ([b43] and [b44]) binding with both the coenzymes and the keto-derivatives.

Regarding proteolytic processes (Fig.4D), such as the ubiquitin proteasome system, several upregulated degradation enzymes ([b8], [b17]*, [b22], and [b24]) play important roles in overall metabolism by reacting homogeneously within transmembrane domains. After 15 and 30 days, relatively downregulated expression of some (Lys and Tyr), but not all (Asn, Asp, and Met), proteolysis-related metabolites was observed (Fig.1A). This observation implies that the amino acids released from proteolytic activities are not being properly utilized during the first 15 days, since the majority of energy is devoted to lignocellulolytic cascades instead of growth. Regarding the metabolic precursors in multiple biosystem, amino acids predicted signs of involvement in glycolysis (3PG), Kreb's cycle (oxaloacetate and *α*-KG), and the pentose phosphate pathway (R5P) (Fig.4E).

In order to reduce the cost of cellulosic carbon, several processes (such as lipolysis and ketogenesis; [b9], [b14], [b18], [b19], and [b36]) for polyhydroxyalkanoate biodegradation were inextricably involved in *P. chrysosporium* metabolism (Fig.4B). The process-related metabolites (especially 2-ethyl-3-hydroxybutyrate, 3-hydroxypropanate, linolenate, and glycerate) showed considerable enrichment after 15 and 30 days. In fungal decomposition, short-chain fatty acids are also believed to play an important role in the effective adsorption of cellulosomes to specific substrates (Bolobova et al. 1994). After Cys exposure and cleavage during microbial biodegradation, the binding modules (especially those that are carbohydrate-specific) are finally acylated with fatty acids. Regarding the role of secreted fatty acids in mediating peroxidase activity, their function may be similar to that of extracellular biosurfactants, which act as a bridge. This is similar to the mechanism through which the activation of hydrolytic enzyme complexes is regulated via intercellular cohesin/dockerin interactions and lipobox modules (Taylor et al. 2006; Weiner et al. 2008). Especially, mitigation of interfacial tension by amphiphilic surfactants (organic compounds) appears to be beneficial for improving % degradation yield (Table4), and these metabolic interactions have been further implicated in catabolic diversity (Fig.4E).

Regarding the absence of nucleotide bases (except for adenosine; Fig.1A), particularly at the later stages (15–30 days), the de novo metabolic system (especially from phosphoribosylpyrophosphate) could improve the efficiency of biosynthesis in fungal biodegradation (Fig.4C). Furthermore, the low expression levels (Fig.2A) of most targets that are involved in ribosomal RNA biogenesis and ribosome processing imply a decrease in recuperative power and fidelity of translation. Regarding the absence (|fold| < 2) of most growth factors, this is particularly abnormal because most ribosomal proteins are thought to enhance the variety of processes related to the linkage between elongation factors and translocational regulation (Petrov et al. 2008). Additionally, although they were generally not significant, the modulation of substrate-specific responses via various growth factors in the *P. chrysosporium* system should still be considered. Lastly, we observed upregulation of BTB/POZ and downregulation of Zn-fingers (especially C_2_H_2_). Even though we observed downregulation of Zn-fingers due to the presence of multiple fingers ([d33]), the GAGA-factor was still predicted to bind to a consensus sequence; selective regulation of these motifs could contribute to elevating the efficiency of biodegradation.

### Cell-to-cell signaling: stress–response pathways

Regulatory messenger systems generally showed a significant decrease under optimized RS culture (or harsh environment) (Figs.2A and 4D and E and Table 2). For example, the influence of protein kinases (PKs; especially [c2], [c12]*, [c14], and [c15]*) are thought to be reduced by the limitation (downregulated expression after 15 days) of target signal transduction (calcium/calmodulin) and several PKs (especially [c12]*) that are induced in cellular damage and tolerance. Especially, downregulation of calcium-dependent PKs can indicate structural destabilization of polysaccharide microfibrils (Yennawar et al. 2006). Furthermore, their potential feedback were broadly predicted as a structural change in intracellular components (Table4). On the other hand, other studies similarly showed that 2 kinases ([c13] and, to a greater extent, [c16]*) are overexpressed under improper growth and phenotypes (in the presence of oncogenes) (Ongkeko et al. 2005). For reference, activated PKs are organically connected with the de novo control system, and therefore can support homeostasis over an entire stages (Ben-Sahra et al. 2013). Furthermore, they may play an important role in inhibiting programmed cell death based on the minimum systems for cellular maintenance.

All defenders (except [c13] and [c16]*) related to detoxification and repair nexuses were expressed at lower levels (Figs.2A and 4D and E and Table 2). For example, Ras signaling involving GTPase superfamilies (|fold| < 2) and downregulated G proteins were observed to promote cell death, and this result is somewhat similar to that of a *Candida* biosystem (Phillips et al. 2006). Regarding growth factor withdrawal and endoplasmic stress, the expression of elongation factors (especially the 1*α* isoform) was shown to offer selective protection against apoptosis (Talapatra et al. 2002). However, the low expression of 1*α* and others (here [d10]*) has been suggested to result in a disproportionally high level of cell proliferation, as shown by current results. Furthermore, under drastic starvation and in the presence of toxic compounds, conserved quorum sensing for the interaction of signals may have particular relevance to promoting synergy for biodegradation. Lastly, the activation of chaperones (especially [c9]*) can universally induce the expression of several heterologous proteins localized to certain regions within the cell. In addition, the positive regulation of *Hsp* genes as well as development-regulated targets shows their impact on chromatin remodeling at specific loci under unexhausted histones ([d15]) (Tsukiyama and Wu 1995). Expectedly, Hsp70 has been reported to be similarly expressed in xenobiotic system (Matsuzaki et al. 2008), similar to ligninolytic system.

## Conclusion

This study is the first to understand the peroxidative lignocellulolysis networks of *P. chrysosporium* based on systemized polyomics approach. In particular, with statistical culture optimization (based on MnP activation) to elucidate synergistic factors, enormous profiles were further reorganized from random information to a regular (or minimum) platform. The current results suggest that the homeostatic pathways involved in the lignocellulolytic system include both those that are directly related to lignin degradation and those that play a supportive role by altering the expression pattern of factors related to central or secondary metabolism, growth signaling, dysfunction repair, and regulatory defense. In the platform based on cell-wall-loosening event, the oxidoreductive mechanisms made various degraders (especially CAZys) more accessible to modified crystalline substrates, and therefore it could ever more improve the % industrial yields. The optimized elucidation of the lignocellulolytic system may allow future metabolic engineering to enhance enzymatic biodegradability of lignocellulose, and thus have a significant impact on improving the thermodynamic efficiency of subsequent bioethanol production.

## Supporting Information

**Table S1.** Primers used for quantitative real-time PCR of selected genes in *Phanerochaete chrysosporium* biosystem.
