# Supplementary material for: Lignocellulose depolymerization occurs via an environmentally adapted metabolic cascades in the wood-rotting basidiomycete Phanerochaete chrysosporium
Source: Microbiologyopen. 2014 Dec 3;4(1):151–66. doi: 10.1002/mbo3.228 (PMC4335982; doi:10.1002/mbo3.228)
Supplement: Supplementary file 1 [file mbo30004-0151-sd1.doc]

**Supporting Information**

Lignocellulose depolymerization occurs via an environmentally-adapted metabolic cascades in the wood-rotting basidiomycete *Phanerochaete chrysosporium*

Jin Seop Bak

*Department of Chemical and Biomolecular Engineering, Advanced Biomass R&D Center, KAIST, 291 Daehak-ro, Yuseong-gu, Daejeon, 305-701, Republic of Korea*

*Address correspondence to J. S. Bak, jsbwvav7@kaist.ac.kr.*

**Supplementary materials and methods**

**General**

Unless otherwise noted, all starting materials were obtained from commercial suppliers and were used without further purification.

**Preparation of starter substrate**

Air-dried RS was milled using a cutting mill (MF 10, IKA, Staufen, Germany) and sieved using 425- and 710-μm mesh sieves (Chung Gye Sang Gong Sa, Seoul, Korea). Crushed RS was dried in a vacuum-drying oven at 45°C for 5 days, and the resulting solid content was found to be 96.6% (w/w). RS was autoclaved at 121°C for 10 min prior to use.

**Preliminary step for target optimization**

In order to control changeable parameters (e.g., growth rate, pH, and protein concentration) and ligninolytic indexes (especially % theoretical yields), I carried out the whole program after the optimization of target enzymes via statistical methodologies. Similar to previously confirmed procedures (ref. 1), a minimum level (1%, w/v) of carbon sources including glucose, galactose, maltose, xylose, arabinose, sucrose, cellobiose, cellulose and starch were checked in the media containing 0.5% (w/v) of yeast extract as a nitrogen source. In addition, the effects of various nitrogen sources at 0.5% (w/v) including yeast extract, peptone, lysine, urea, (NH_4_)_2_SO_4_ and NaNO_3_ were tested in media containing 1% (w/v) of glucose as a carbon source. Based on the target carbon (here glucose) and nitrogen source (here yeast extract) from the preliminary experiments, fungal medium was further optimized using Placket-Burman design with other components such as KH_2_PO_4_, CaCl_2_, MgCl_2_, FeSO_4_ and vitamin solution. As the predominant variables was identified as yeast extract, CaCl_2_ and KH_2_PO_4_ by PBD, these medium components were subsequently optimized using the Box-Behnken design. Finally, yeast extract, KH_2_PO_4_ and CaCl_2_ were determined as 7.290, 3.087 and 0.034 g/L, respectively. Under the optimized condition, the predicted activity of heam peroxidase was approximately 2,741 U/L. Its actual activity from the experiments was confirmed in good agreement (~ 2,700 U/L) with the predicted values.

**Assay of lignocellulolytic targets in extracellular biosystem**

Regarding a unit (IU, international units) of all enzymes, the enzyme activity was defined as an amount of enzyme that releases 1 μmol of reducing target (monomer equivalent) per min. Similar to previously used methods (ref. 1, ref. 2), the activity of extracellular heam peroxidase (especially MnP) was checked by detecting the formation of a Mn (III)-malonate unit at 270 nm after incubating mixtures containing 0.1 mM H_2_O_2_ and 0.2 mM MnSO_4_ at 30°C by time intervals. Furthermore, the activity of cell-bound peroxidase was confirmed as a sum of intact cell-bound and cell envelope-bound activity. In order to check the other peroxidase (i.e., LiP), the oxidation of VA to veratraldehyde was observed simultaneously at 310 nm (ref. 3, ref. 4). Regarding peroxidative cytochrome enzymes, the activity of GLOX was measured by monitoring the formation of H_2_O_2_ using a modified peroxidase-coupled assay (ref. 3, ref. 5, ref. 6). Also, AAO was analyzed by incubating the mixture containing 2.5 mM VA in 20 mM sodium succinate (pH 3–4) at 30 ^o^C, and the oxidation of VA was checked at 310 nm (ref. 3, ref. 7, ref. 8). The peroxidative activity of multicopper oxidase was checked by monitoring the generation of ferric ion at 315 nm. The level of oxidation was analyzed using 5 mM ferrous ammonium sulphate in sodium acetate (pH 5, 100 mM) at 30 ^o^C (ref. 9). Here, ferroxidase activity was limited by NaF. The activity of P450-oxidoreductase was predicted based on its degree to reduce cytochrome c using NADPH by monitoring the change in absorbance at 550 nm (ref. 10). Regarding the glutathione mechanism, glutathione/glutathione disulfide ratio (it was expressed as “[glutathione − (2 glutathione disulfide)]/glutathione disulfide”) was examined by using the OxisResearch assay kit (Cat no. 21040, Portland, OR) according to the manufacturer's instructions. This reaction was detected 2-nitro-5-thiobenzoate anion (reacts with glutathione) at 412 nm. Additionally, gluthathione reductase was assayed by monitoring the generation of the anion. Especially, target samples (reacted with gluthathione reductase) can induce some glutathiones, and then was added 1-methyl-2-vinylpyridinium trifluoromethanesulfonate in order to scavenge the glutathiones. As a result, the glutathione disulfide was changed to glutathione with NADPH. For reference, if you were to be monitored the coupled recycling reaction, you could predict the activity of glutathione peroxidase. The unit of glutathione peroxidase was indicated as the oxidation of 1 μmol NADPH per min. Peroxidative catalase activity was checked by observing the change of H_2_O_2_ at 240 nm. The activity of superoxide dismutase (especially Cu/Zn-superoxide dismutase) was based on hematoxylin autoxidation, which monitored the change of hematoxylin in alkaline solution for 1 min at 560 nm (ref. 11). Furthermore, because superoxide dismutase (especially Mn-superoxide dismutase) is very sensitive to H_2_O_2_, its activity can be confirmed under the presence of KCN (5 mM).

In extracellular cellulolytic process for bioethanol production, the activities of key CAZys were indirectly checked by industrial indexes (here digestibility and fermentation yield) as well as metabolomic profiles; this is because the final products (by key CAZys) are monomeric sugars (especially glucose) in cellulose biodegradation. The sugar digestibility (Eq. 1) and ethanol yield (Eq. 2) was indicated as a percentage of the theoretical maximum of substrate obtained from raw material (i.e., unpretreated substrate). Especially, the actual activity of major CAZys (especially β-glucosidase and CDH) was analyzed based on the generally confirmed method (ref. 12). First, the power of β-glucosidase was measured by monitoring the separation of p-nitrophenyl from p-nitrophenyl-β-D-glucoside at 400 nm for 5 min. Next, extracellular CDH was assayed by the reduction of *cytochrome c* (12.5 μM) at 550 nm in the presence of cellobiose (100 μM). Additionally, *cytochrome c* power of CDH was confirmed via 2,6-dichlorophenolindophenol activity (1: 1.54 unit).

$\text{Glucose yield (\% theoretical maximum) }=\text{ }\frac{\text{g of glucose by HPLC}}{\text{g of glucan}\times1.1}\times100$ (Eq. 1)

$\text{Ethanol yield (\% theoretical maximum) } = \text{ }\frac{\text{g of ethanol}}{\text{g of glucan }\times1.1\times0.511}\times100$ (Eq. 2)

In extracellular hemicellulolysis (especially by xylanase), 1 mL mixture containing 0.5 mL of diluted supernatant of fungal biodegradation broth and 0.5 mL of 1–2% birchwood xylan (Sigma-Aldrich, St. Louis, MO) in 0.05 M sodium citrate buffer (pH 4.8) was analyzed at 50 °C for 30 min, and the reducing sugars were checked by the dinitrosalicylic acid protocol (http://www.nrel.gov/biomass/analytical_procedures.html) (ref. 13).

**RNA amplification and quality check**

Amplified RNA was obtained using a MessageAmp II-Biotin Enhanced Single Round Amplification Kit (Ambion, Austin, TX) (ref. 14). In order to solve common problems (e.g., low quality, loss, and contamination) during the purification of RNA, an RNeasy kit (Qiagen, Valencia, CA) was used simultaneously. Finally, RNA purity was determined by measuring the ratio of absorbance at 230, 260, and 280 nm in a NanoDrop spectrophotometer (ND**-**1000, Thermo Fisher Scientific, Wilmington, DE).

**Probe matching test**

First of all, because simultaneous biodegradation system (or enzymatic hydrolysis and fermentation) was carried out in downstream cascades regarding lignocellulolytic metabolism, the selection of efficient organism was needed for enhancing the degradation efficiency. Thus, *P. chrysosporium* (ATCC 32629) via process optimization (ref. 1) instead of RP78 (ATCC 20696) was used. Although the target organism is not sequenced by JGI or another institute, the similarity is very high with each other. I tried to check its validation as initial confirmation which RP78-probes (derived from RP78 genome map in JGI) was designed and used for matching (or reference) on target organism, and then confirmed a sign of “no problem” in significant expression, except for some genes (i.e., below 1%). For reference, the procedure of probe design is not complicate and difficult in order to verify some errors on the array. I ignored the uncertain errors and unknown targets (by JGI), and then significant targets could select for array-chip analysis. Furthermore, one more time, the designs were confirmed by a change of expression patterns in protein level.

**Microarray and data analysis**

The hybridization was carried out at 45^o^C for 16 h with gentle rotation, after which the arrays were washed as following: 6× saline-sodium phosphate-ethylenediaminetetraacetic acid (SSPE) and 0.05% (w/v) Tween-20 for 5 min, 3× SSPE and 0.05% Tween-20 for 1 min, 0.5× SSPE and 0.05% Tween-20 for 1 min, and 2× PBS and 0.1% Tween-20 for 1 min. Biotin labeling was conducted with gentle rotation for 30 min. The arrays were then washed with 2× PBS, 0.1% Tween-20 for 1 min, and 2× PBS for 1 min. A LifterSlip (Erie Scientific, Portsmouth, NH) was laid at an angle onto the microarray (so that it was centered over the semiconductor area). First, the imaging solution was placed on one side of the LifterSlip. The slip was then slowly lowered to avoid incoming air bubbles. The hybridized arrays were scanned at PMT voltages of 500–700 with a pixel size of 5 and a focus position of 130 using a GenePix 4200A microarray scanner (Axon Instruments, Union City, CA). After completing the scan, the images were visually examined and used for data extraction (https://webapps.combimatrix.com). After the data extraction, the background was calculated for individual samples using factory-built control probes with low intensities (5–30%), and their median signal intensities were referenced for background subtraction. Array datum for each sample was processed by global normalization using probes with signals greater than zero. A total of 5,621 probes (actually > 10,000 genes; except for unknown genes and overlaps) with signals lower than 60,000 (i.e., saturation value), and those signals higher than 5% of the lowest signal of each sample were subjected to final analysis. Student’s *t*-test was applied to determine differentially expressed sets of genes across experimental groups—control and RS. Statistical significance was adjusted using the Benjamini-Hochberg FDR multiple testing correction (ref. 15). Avadis Prophetic ver. 3.3 (Strand Genomics, Bangalore, India; http://avadis.strandgenomics.com/) was used as the statistical software.

**Complementary qRT-PCR**

Aliquots of cDNA (10 μl) were added to 384-well microtiter plates for quantitative real-time PCR (qRT-PCR), which was performed with an ABI-PRISM 7900HT Sequence Detection System (Applied Biosystems, Foster City, CA). The cDNA synthesis was performed using 500 ng RNA at 42°C, and the resultant cDNA was diluted 1:2 prior to use for the qRT-PCR. For PCR, 90 nM of primer(s), 250 nM of fluorescence-labeled TaqMan probe, and 5 μl of Universal Master Mix (Applied Biosystems) were used. Finally, 2 μl of cDNA template was added to the reaction mixture. A combination of 5 primers and TaqMan probes was designed on the basis of the genome sequences from the *P. chrysosporium* database by the DOE-JGI. Amplification was performed at 95°C for 10 min for template denaturation, followed by 40 cycles at 95°C for 0.25 min and 60°C for 1 min. All samples were amplified in triplicates and the data were analyzed using sequence detector software (Applied Biosystems). The conventional PCR efficiency of target genes was reasonably high with 2^n^ copies/cycle. The relative quantities of mRNA of selected genes were calculated by the 2^–∆∆Ct^ method (ref. 16).

**Protein extraction and 2-DE**

After six biological replicates, *P. chrysosporium* mycelial pellets (obtained from 15-day cultures) were washed twice in ice-cold PBS and sonicated for 10 sec with a Bandelin Sonoplus HD 200 (Bandelin Electronic, Berlin, Germany). The pellets were then incubated overnight at 4°C after the addition of 10 ml of 10% (w/v) trichloroacetic acid per cell mass (mg), and they were centrifuged at 15,000×*g*. Acetone was added to the resultant pellets, and the mixture was incubated for 1 h at 4°C. After centrifugation at 15,000×*g* for 1 h at 15°C, the dried pellets were dissolved in a sample lysis solution [7 M urea, 2 M thiourea containing 4% (w/v) CHAPS, 1% (w/v) DTT, 2% (v/v) pharmalyte, and 1 mM benzamidine]. Extraction process was carried out by vortexing for 1 h at room temperature. After centrifugation at 15,000×*g* for 1 h at 15°C, the soluble fraction was used for two-dimensional gel electrophoresis (2-DE). For the normalization of protein loading in each sample, the total protein concentration was determined by a Bradford assay (ref. 17). IPG dry strips (pH 4–10 NL, 24 cm, Amersham Biosciences, NJ) were equilibrated for 12–16 h with 7 M urea and 2 M thiourea containing 2% (w/v) CHAPS, 1% (w/v) DTT and 1% (w/v) pharmalyte, and then were loaded with 200 µg of sample proteins. IEF was performed at 20°C using a Multiphor II electrophoresis unit (Amersham Biosciences). For the IEF as the first dimension separation, the voltage was linearly increased from 150 to 3500 V over 3 h for the sample entry, and was maintained at 3500 V for a total of 96000 Vh. Prior to the second dimension, the strips were incubated in an equilibration buffer [50 mM Tris-HCl (pH 6.8), 6 M urea, 2% (w/v) SDS, and 30% (w/v) glycerol] for 10 min and were washed with 1% (w/v) DTT for the reduction of disulfide bridges. The strips were also incubated for 10 min in an equilibration buffer solution and were washed with 2.5% (w/v) iodoacetamide for the alkylation of free cysteines. Equilibrated strips were inserted onto SDS-PAGE gels (20 × 24 cm, 10–16%). The SDS-PAGE was performed using a Hoefer DALT 2D system (Amersham Biosciences). The 2-DE gels were run at 20°C for 1700 Vh, and then were stained with CBB G250. Quantitative analysis of digitized images was carried out using PDQuest software ver. 7.0 (BioRad, Hercules, CA). Protein spots with significant expression variation (|fold| > 2 and *P* < 0.05) under RS as compared to the expression level of the control were selected for analysis.

**Protein ID and data analysis**

The selected spots in 2-DE gels were enzymatically digested using modified porcine trypsin method (ref. 18). Gel segments were washed with 50% (w/v) aqueous ACN to remove SDS, salt, and stain. They were subsequently dried to remove solvent and were rehydrated with trypsin (8–10 ng/μl) before the incubation for 8–10 h at 37°C. The proteolytic reactions were terminated by adding 5 μl of 0.5% TFA. Tryptic peptides were recovered by mixing the aqueous phase from several extractions of the gel pieces with 50% (w/v) ACN. The peptide mixture was desalted (and concentrated) using micro C_18_ZipTips (Millipore, Bedford, MA) and eluted in 1–5 μl ACN. An aliquot of this solution was mixed with an equal volume of a saturated solution of CHCA in 50% (w/v) ACN, and 1 μl of the mixture was spotted onto a target plate. Protein analysis (by chemically assisted fragmentation) was performed using an Ettan MALDI-TOF (Amersham Biosciences). Peptides were evaporated with N_2_ laser at 337 nm by using a delayed extraction approach. They were accelerated with a 20-kV injection pulse for the analysis of flight time. Each spectrum showed the cumulative average values of 300 laser shots. ProFound (http://129.85.19.192/profound_bin/WebProFound.exe) was used for protein ID by PMF. The spectra were calibrated with trypsin auto-digestion ion peaks (at m/z 842.510 and 2211.104) used as the internal standards. To enhance the ID accuracy, the protein spots were simultaneously analyzed using either a Voyager-DE STR MALDI-TOF (Applied Biosystems) or a 4700 Proteomics Analyzer MALDI-TOF/TOF (Applied Biosystems). When necessary, the samples were first desalted using the C_18_ZipTips and the peptides were eluted directly with 5 mg/mL of CHCA in 60% (w/v) ACN/0.1% (w/v) TFA onto a MALDI plate. All the MS spectra were simultaneously recorded in positive reflector mode. For MS data, 200 and 1000 shots were accumulated for each spectrum (obtained from the Voyager-DE STR and the TOF/TOF, respectively). All MS/MS data from the TOF/TOF were acquired using the default 1 kV MS/MS method installed by the manufacturer. Proteins from the MS/MS data were identified using the Protein Prospector (http://prospector.uscf.edu), the Proteomic Solution 1 system (Applied Biosystems), and the DOE-JGI database. A total of 150 spectral peaks were systematically submitted for database searching against a small database composed of the sequences of the protein standards alone.

**Fungal metabolome extraction**

*P. chrysosporium* cells (separated from the fermented medium) were executed as following: 5 ml of sample was injected into 26 ml of cold solution containing 60% (v/v) methanol (or chloroform-methanol (2 : 1) as much as possible) buffered with 70 mM HEPES at pH 7.5. This was then maintained at -40°C in a dry ice/ethanol bath. After cooling for 3 min, the mixture was centrifuged at 5,000×*g* for 5 min at 0°C. After the centrifugation, the mixture was maintained at -20°C. Fungal cell mass was determined to be approximately 30 mg dry weight after the drying using a vacuum-drying oven at 55°C. Endometabolome was directly extracted from the pellets in 5 ml of 75% (v/v) boiling absolute ethanol buffered with 0.25 M HEPES at pH 7.5, and this solution was incubated for 3 min at 80°C. After cooling the mixture on ice for 3 min, it was dehydrated using Speed Vac Plus device (SC110A, Savant Instruments, Holbrook, NY) at 25°C. For exometabolome analysis, after filtration through a 0.2-μm PVDF filter (Whatman), the supernatant from fungal culture broths was evaporated to dryness under vacuum with the Speed Vac Plus at 25°C. After the vacuum drying, both metabolome samples were resuspended in 80 μl of methoxyamine hydrochloride solution in pyridine (2 g/100 ml), and incubated for 90 min at 30°C. Eighty milliliter of *N*-Methyl-*N*-(trimethylsilyl)trifluoroacetamide was then added to each sample, followed by incubation for 30 min at 37°C. Here sorbose (62.5 nmol per sample) was used as the internal standard. Finally, all samples are stored in a freezer (-20°C).

**GC-MS operation and** **metabolite identification**

The total ion chromatograms (TICs) using GC-MS was performed to analyze the expression patterns of *P. chrysosporium* metabolome on RS as compared to those of the control. Helium gas was used as the mobile phase at 1 ml He/min. The injection volume was 1 μl, and the split ratio was 1:100. The GC-MS was operated in scan mode in the mass range of 50–550 amu. The gradient used for the GC-MS analysis was as follows: 70°C for 5 min, 10°C/min to 179°C, 0.5°C/min to 180 °C with holding for 2 min, 10°C/min to 220°C with holding for 1 min, 2.5°C/min to 265°C with holding for 1 min, 10°C/min to 280°C with holding for 1 min, 1°C/min to 290°C, and 10°C/min to 300°C. More than 300 peaks were obtained, and then predominant extracellular 190 metabolites confirmed by spectral matching against the database from the National Institute of Standards and Technology research library (http://www.nist.gov/srd/nist1a.htm); approximately 80 compounds have been identified in at least 75% of subjects in all profiles. To minimize analytical errors, the retention times were corrected using internal reference compounds (ref. 19). The integrated area of each peak obtained from the TIC normalized using authentic standards were utilized for the quantitative comparison of each metabolite.

**Determining metabolic fluxes from multiple omics and downstream data**

From the relative fold-change between control and optimized condition, the probable course of lignocellulolytic pathway (Fig. 3E in main text) can be predicted, due to the fact that a large fold-change indicates a high rate and quantity of production. A statistical approach to these integrated profiles revealed that the global change in substrate-specific regulation and the mainstream cascade correlates directly with the change in enzyme expression levels. The correlation was predicted and verified based on results from the literature, by testing byproducts (e.g., fermentable sugars and intermediates or precursors) as alternative substrates (guaranteeing reversed fluxes in biodegradation), and via certain gene expression perturbations.

For novel organisms or regulatory systems without any known information on metabolic pathways, determining the order of metabolites within the whole system may be highly difficult if the observed fold-changes are considerable similar. In this case, the addition of regulomics studies containing kinetics can provide sufficient information in order to reach a valid conclusion regarding the path and direction of the underlying mechanism. However, flux data or its *in silico* models were not necessary for the purpose of current research, since the details of the lignocellulolytic system and some of the pathways have already been partially characterized.

**Supplementary References**

1. Bak, J. S., Ko, J. K., Choi, I. G., Park, Y. C. et al., Fungal pretreatment of lignocellulose by *Phanerochaete chrysosporium* to produce ethanol from rice straw. *Biotechnol. Bioeng.* 2009, *104*, 471–482.
2. Perie, F., Gold, M., Manganese regulation of manganese peroxidase expression and lignin degradation by the white rot fungus *Dichomitus squalens*. *Appl. Environ. Microbiol.* 1991, *57*, 2240–2245.
3. Teunissen, P. J. M., Field, J. A., 2-Chloro-1,4-dimethoxybenzene as a novel catalytic cofactor for oxidation of anisyl alcohol by lignin peroxidase. *Appl. Environ. Microbiol.* 1998, *64*, 830–835.
4. Tien, M., Kirk, T. K., Lignin-degrading enzyme from *Phanerochaete chrysosporium*: purification, characterization, and catalytic properties of a unique H_2_O_2_-requiring oxygenase. *Proc. Natl. Acad. Sci. USA* 1984, *81*, 2280–2284.
5. Kersten, P. J., Kirk, T. K., Involvement of a new enzyme, glyoxal oxidase, in extracellular H_2_O_2_ production by *Phanerochaete chrysosporium*. *J. Bacteriol.* 1987, *169*, 2195–2201.
6. Orth, A. B., Denny, M., Tien, M., Overproduction of lignin-degrading enzymes by an isolate of *Phanerochaete chrysosporium*. *Appl. Environ. Microbiol.* 1991, *57*, 2591–2596.
7. Bourbonnais, R., Paice, M. G., Veratryl alcohol oxidases from the lignin-degrading basidiomycete *Pleurotus sajor-caju*. *Biochem. J.* 1988, *255*, 445–450.
8. Muheim, A., Waldner, R., Leisola, M. S. A., Fiechter, A., An extracellular aryl-alcohol oxidase from the white-rot fungus *Bjerkendera adusta*. *Enzyme Microb. Technol.* 1990, *12*, 204–209.
9. Hassett, R. F., Yuan, D. S., Kosman, D. J., Spectral and kinetic properties of the Fet3 protein from *Saccharomyces cerevisiae*, a multinuclear copper ferroxidase enzyme. *J. Biol. Chem.* 1998, *273*, 23274–23282.
10. Subramanian, V., Doddapaneni, H., Syed, K., Yadav, J. S., P450 Redox enzymes in the white rot fungus *Phanerochaete chrysosporium*: gene transcription, heterologous expression, and activity analysis on the purified proteins. *Curr. Microbiol.* 2010, *61*, 306–314.
11. Martin, J. P., Dailey, M., Sugarman, E., Negative and positive assays of superoxide dismutase based on hematoxylin autoxidation. *Arch. Biochem. Biophys.* 1987, *255*, 329–336.
12. Bao, W., Lymar, E., Renganathan, V., Optimization of cellobiose dehydrogenase and β-glucosidase production by cellulose-degrading cultures of *Phanerochaete chrysosporium*. *Appl. Microbiol. Biotechnol.* 1994, *42*, 642–646.
13. Miller, G. L., Use of dinitrosalicylic acid reagent for determination of reducing sugar. *Anal. Chem.* 1959. *31*, 426–428.
14. Van Gelder, R. N., von Zastrow, M. E., Yool, A., Dement, W. C. et al., Amplified RNA synthesized from limited quantities of heterogeneous cDNA. *Proc. Natl. Acad. Sci. USA* 1990, *87*, 1663–1667.
15. Benjamini, Y., Hochberg, Y., Controlling the false discovery rate: a practical and powerful approach to multiple testing. *J. R. Statist. Soc.* 1995, *B57*, 289–300.
16. Livak, K. J., Schmittgen, T. D., Analysis of relative gene expression data using real-time quantitative PCR and the 2(-Delta Delta C(T)) Method. *Methods* 2001, *25*, 402–408.
17. Bradford, M. M., A rapid and sensitive method for the quantitation of microgram quantities of protein utilizing the principle of protein-dye binding. *Anal. Biochem.* 1976, *72*, 248–254.
18. Shevchenko, A., Wilm, M., Vorm, O., Mann, M., Mass spectrometric sequencing of proteins silver-stained polyacrylamide gels. *Anal. Chem.* 1996, *68*, 850–858.
19. Fiehn, O., Kopka, J., Trethewey, R. N., Willmitzer, L., Identification of uncommon plant metabolites based on calculation of elemental compositions using gas chromatography and quadrupole mass spectrometry. *Anal. Chem.* 2000, *72*, 3573–3580.

**Supplementary** **table legends**

**Table S1.** Primers used for quantitative real-time PCR of selected genes in *P. chrysosporium* biosystem.

**Table S1. Primers used for quantitative real-time PCR of selected genes in *P. chrysosporium* biosystem.**

| No./Interpro ID | Putative function ^a^ | Primer sequence (forward /reverse) | Amplicon  (bp) | Fold change ^b^ | |
| --- | --- | --- | --- | --- | --- |
|  |  |  |  | Arrary | qRT-PCR |
| [a3]/IPR000254 | Cellulose-binding domain | GTGTCTTCGACTAGCACAAGCA  /TCCTCGACTGCACGAATGAAG | 67 | 2.9 ± 0.5 | 7.2 ± 1.2 |
| [a8]/IPR002016 | Haem peroxidase | CTCGGCGCGGTGACT  /GGTCTCCAGGGCACGT | 83 | 3.8 ± 1.0 | 2.5 ± 0.6 |
| [a9]/IPR005829 | Sugar transporter | GTGAGAAGCTCAAAGACTTTGCA  /GAAGCTGAGGCCCATGGA | 80 | 14.7 ± 2.6 | 1.4 ± 0.2 |
| [b35]/IPR000608 | Ubiquitin-conjugating enzyme | GCCACGAAGCACTTGCA  /CGCCTTGCGGGTGATGA | 83 | -2.1 ± 0.5 | -2.4 ± 0.8 |
| [d8]/IPR007216 | Cell differentiation/sexual development, Rcd1-like | ACACGTCCGTTTGAATATCTTCGT  /CCGTACTGTTGTCATTTTGCTTGA | 79 | -3.3 ± 0.7 | -12.9 ± 2.9 |

^a^ The functions of the selected genes of *P. chrysosporium* were marked and assigned by the US Department’s Joint Genome Institute.

^b^ Ratios of rice straw culture to the control without the substrate.
